# Tumor-dose-rate variations during robotic radiosurgery of oligo and multiple brain metastases

**DOI:** 10.1007/s00066-020-01652-6

**Published:** 2020-06-25

**Authors:** Maria-Lisa Wilhelm, Mark K. H. Chan, Benedikt Abel, Florian Cremers, Frank-Andre Siebert, Stefan Wurster, David Krug, Robert Wolff, Jürgen Dunst, Guido Hildebrandt, Achim Schweikard, Dirk Rades, Floris Ernst, Oliver Blanck

**Affiliations:** 1 Department of Radiation Oncology, University Medicine Rostock, Rostock, Germany; 2 Saphir Radiosurgery Center Frankfurt and Northern Germany, Guestrow, Germany; 3 Department of Radiation Oncology, Karl-Lennert-Krebscentrum Nord, University Medical Center Schleswig-Holstein, Campus Kiel, Arnold-Heller-Straße 3, Haus 50, 24105 Kiel, Germany; 4 Strahlenklinik, University Hospital Essen, Hufelandstr. 55, Essen, Germany; 5 Institute for Robotics and Cognitive Systems, University of Luebeck, Luebeck, Germany; 6 Department of Radiation Oncology, University Medical Center Schleswig-Holstein, Luebeck, Germany; 7 Department of Radiation Oncology, University Medicine Greifswald, Greifswald, Germany; 8 Department of Neurosurgery, University Hospital Frankfurt, Frankfurt, Germany

**Keywords:** Radiation biology, CyberKnife, Stereotactic radiosurgery, SRS

## Abstract

**Purpose:**

For step-and-shoot robotic stereotactic radiosurgery (SRS) the dose delivered over time, called local tumor-dose-rate (TDR), may strongly vary during treatment of multiple lesions. The authors sought to evaluate technical parameters influencing TDR and correlate TDR to clinical outcome.

**Material and methods:**

A total of 23 patients with 162 oligo (1–3) and multiple (>3) brain metastases (OBM/MBM) treated in 33 SRS sessions were retrospectively analyzed. Median PTV were 0.11 cc (0.01–6.36 cc) and 0.50 cc (0.12–3.68 cc) for OBM and MBM, respectively. Prescription dose ranged from 16 to 20 Gy prescribed to the median 70% isodose line. The maximum dose-rate for planning target volume (PTV) percentage *p* in time span *s* during treatment (TDR_s,p_) was calculated for various *p* and *s* based on treatment log files and in-house software.

**Results:**

TDR_60min,98%_ was 0.30 Gy/min (0.23–0.87 Gy/min) for OBM and 0.22 Gy/min (0.12–0.63 Gy/min) for MBM, respectively, and increased by 0.03 Gy/min per prescribed Gy. TDR_60min,98%_ strongly correlated with treatment time (ρ = −0.717, *p* < 0.001), monitor units (MU) (ρ = −0.767, *p* < 0.001), number of beams (ρ = −0.755, *p* < 0.001) and beam directions (ρ = −0.685, *p* < 0.001) as well as lesions treated per collimator (ρ = −0.708, *P* < 0.001). Median overall survival (OS) was 20 months and 1‑ and 2‑year local control (LC) was 98.8% and 90.3%, respectively. LC did not correlate with any TDR, but tumor response (partial response [PR] or complete response [CR]) correlated with all TDR in univariate analysis (e.g., TDR_60min,98%_: hazard ration [HR] = 0.974, confidence interval [CI] = 0.952–0.996, *p* = 0.019). In multivariate analysis only concomitant targeted therapy or immunotherapy and breast cancer tumor histology remained a significant factor for tumor response. Local grade ≥2 radiation-induced tissue reactions were noted in 26.3% (OBM) and 5.2% (MBM), respectively, mainly influenced by tumor volume (*p* < 0.001).

**Conclusions:**

Large TDR variations are noted during MBM-SRS which mainly arise from prolonged treatment times. Clinically, low TDR corresponded with decreased local tumor responses, although the main influencing factor was concomitant medication.

## Introduction

Stereotactic radiosurgery (SRS) is considered standard therapy for oligo (1–3) brain metastases (OBM) [1–3]. The treatment of multiple (>3) brain metastases (MBM) with SRS vs. whole-brain radiotherapy (WBRT) is subject to debate [4–6]. Some studies suggested that not the number of metastases but clinical factors like performance status, histology and extra-cranial manifestations are leading survival factors [6, 7]. Furthermore, for melanoma (MLA), non-small-cell-lung-cancer (NSCLC), breast-cancer (BC) and renal-cell carcinoma (RCC) and concomitant with targeted therapy or immunotherapy (TT/IT) there is now sufficient evidence of efficacy and safety for MBM-SRS [8–11].

Nonetheless, technical challenges for MBM-SRS remain which include dose delivery over time to all tumor cells and simultaneous minimization of healthy brain dose. To achieve this, precise treatment delivery with small safety margins and numerous beam directions and/or isocenters to create steep non-intersecting dose gradients are necessary [12]. However, the treatment complexity will automatically increase treatment time, which in turn will decrease the dose delivered to certain tumor cells within certain time spans. This phenomenon is called tumor-dose-rate (TDR) effects, which are loco-regional effects derived from dose-accumulation differences during treatment. Ultimately, the dose to most tumor cells (e.g., 98% [13, 14]) in the gross tumor volume (GTV) or planning target volume (PTV) will reach the planned minimum dose (e.g., 16–20 Gy). However, for the dose delivered in, e.g., half the treatment time the situation may be different when some cells may have already received the planned dose while other cells may have not even reached the dose by far.

Biologically, the TDR phenomenon translates into the possibility of tumor cells receiving dose over longer periods of time to repair radiation-induced DNA damage during prolonged treatment. This has been reported for healthy cell lines [15, 16]; however, for tumor cells the reports are somewhat inconclusive. One in-vitro analysis found no differences in tumor cell responses for long-pulsed vs. short-burst dose delivery [17]. Another study even found that intermittent irradiation significantly reduced the survival of glioblastoma cells compared with continuous irradiation [18]. On the other hand, TDR effects have been reported for Gamma Knife SRS (Elekta, Stockholm, Sweden), where changes in treatment plan complexity can influence treatment time and hence biological equivalent dose (BED) when including treatment time components [19, 20].

In contrast, the main problem for CyberKnife SRS (Accuray, Sunnyvale, USA) arises from step-and-shoot delivery and regional-dose-rate effects have been reported for potential treatments of atrial fibrillation where healthy cells are targeted and dose-rate differences are expected to manifest clinically [21]. Regardless, there is a clear lack in clinical data for in-vivo tumor configurations and the authors attempted to overcome this shortcoming by investigating the TDR phenomenon for CyberKnife MBM-SRS.

## Material and methods

### Patient and treatment characteristics

All MBM-SRS cases treated between 11/2011 and 08/2017 were retrospectively selected. This group consisted of 18 patients with 143 metastases treated in 22 SRS sessions (4–20 metastases/session). After adding the OBM-SRS treatments of the same patients (*n* = 3), OBM-SRS cases were randomly added from the matched tumor volume and dose range treatment database until 2:1 session split between MBM and OBM was reached. The OBM cohort then consisted of eight patients with 19 brain metastases treated in 11 SRS sessions (1–3 metastases/session). Overall, 23 patients with 162 brain metastases treated in 33 SRS sessions were analyzed. Primary tumor histology was NSCLC (*n* = 10), MLA (*n* = 8), BC (*n* = 4) and RCC (*n* = 1).

Median single and cumulative PTV/session (GTV + 0–1 mm) were 0.10 cc (0.01–4.64 cc) and 1.77cc (0.17–13.66 cc) for MBM and 0.49cc (0.11–3.58 cc) and 0.83 cc (0.11–4.07 cc) for OBM, respectively. PTV D_98%_ ranged from 16 to 20 Gy (median, OBM = 20 Gy and MBM = 18 Gy) prescribed to the median 70% (59–83%) isodose line. For all cases between one and four fixed cylindrical collimators of 5–15 mm diameter were used depending on size, shape, location and number of metastases. Treatment plan optimization was performed according to best practice guidelines [22, 23], which included GTV mean dose optimization [24] wherever necessary and dedicated minimization of healthy brain volume receiving 3–12 Gy [25] in a trade-off against treatment time. Session treatment times as captured by log files were median 104 min (36–226 min) for MBM and 60 min (23–123 min) for OBM, respectively.

Additional WBRT was given prior to nine sessions (40.9%) for MBM and five sessions (45.4%) for OBM, respectively, yet not within 3 months of SRS. Additional TT/IT [8], chemotherapy and no additional therapy within 30 days of SRS was given for 11, 12 and 10 SRS sessions, respectively (Table 1).

**Table 1:** Patient and treatment characteristics

			Total	%
Patients			23	100
	OBM group		5	21.7
	MBM group Both groups		15 3	65.2 13.1
Lesions			162	
	OBM group		19	11.7
	MBM group		143	88.3
SRS sessions			33	100
	OBM group		11	33.3
	MBM group		22	66.6
Lesions/session	1		6	18.2
	2–3		5	15.1
	4		9	27.3
	5–6		7	21.2
	7–9		3	9.1
	≥10		3	9.1
Gender	Male		7	30.4
	Female		16	69.6
Age	Median (range) in years		63	(39–82)
Single PTV	OBM group	Median (range) in cm^3^	0.49	(0.11–3.58)
	MBM group	Median (range) in cm^3^	0.10	(0.01–4.64)
Cumulative PTV	OBM group	Median (range) in cm^3^	0.83	(0.11–4.07)
	MBM group	Median (range) in cm^3^	1.77	(0.17–13.66)
PTV D_98%_	OBM group	Median (range) in Gy	20	(18–20)
	MBM group	Median (range) in Gy	18	(16–20)
PTV D_max_	OBM group	Median (range) in Gy	27.0	(24.1–29.0)
	MBM group	Median (range) in Gy	25.8	(23.1–29.0)
Prescription isodose	OBM group	Median (range) in %	73	(68–83)
	MBM group	Median (range) in %	70	(59–78)
Beams/lesion	OBM group	Median (range)	118	(69–254)
	MBM group	Median (range)	36	(8–139)
MU/lesion	OBM group	Median (range)	11877	(7482–22,560)
	MBM group	Median (range)	5569	(426–20,755)
Treatment time	OBM group	Median (range) in min	60	(23–123)
	MBM group	Median (range) in min	104	(36–226)
WBRT before SRS	OBM group		5	45.5
	MBM group		9	40.9
Concomitant therapy	OBM group	TT/IT	4	36.4
		Chemotherapy	4	36.4
		No therapy	3	27.2
	MBM group	TT/IT	7	31.8
		Chemotherapy	8	36.4
		No therapy	7	31.8

*OBM* Oligo (1–3) brain metastases, *MBM* multiple (≥4) brain metastases, *SRS* stereotactic radiosurgery, *PTV* planning target volume, *MU* monitor units, *WBRT* whole brain radiotherapy, *TT/IT* targeted therapy/immunotherapy

### Tumor-dose-rate calculation

To calculate TDR, the treatment data (system calibration files, treatment planning files including planning CT and beam configurations, and treatment log files including specific beam-on times) was imported into an in-house planning system (eCKP, version 2.1) [21, 26]. The authors then recalculated the accumulative dose for each PTV voxel for every minute over the whole treatment course.

For time span *s* (in minutes) and voxel *v* and any time point *t* (in minutes) during treatment the TDR is defined as:

1 $$TDR\left(s,v,t\right)=\frac{\textit{ACCDOSE}\left(v,t\right)-\textit{ACCDOSE}\left(v,t-s\right)}{s}$$

(*in Gy/min*)where *ACCDOSE(v,t)* is the accumulative dose of voxel *v* at time point *t*. For time span *s* and each voxel *v* the maximum TDR is defined as:

2 $$TDR(s,v)=\mathit{\max }_{s\leq t\leq T}\left(TDR\left(s,v,t\right)\right)$$

where T is the total treatment time (in minutes). Since it is difficult to assess each voxel separately, the authors further calculated the maximum TDR for a percentage of the PTV (i.e., 50% or 98%). In other words, they calculated the TDR for which 50% or 98% of the voxels reach at least a certain TDR at any time span during treatment. For time span *s* and PTV percentage *p* (range, 0–1) the TDR is defined as:

3 $$TDR\left(s,p\right)=TDR\mathrm{'}(s,\left| PTV\right| *\left(1-p\right))$$

with *TDR′(s,v*_*i*_*)* ≤ *TDR′(s,v*_*i*_ _*+*_ _*1*_*) *and *|PTV|* is the amount of PTV voxels *v*. If the TDR is sorted according to its values as demanded by Eq. 3 one can also display the TDR as a tumor-dose-rate histogram for any given time span *s*. Specific clinical relevant time spans (*s* *=* *20, 40, 60, 80, 100, 120* *min*) [15–20] and PTV percentages (*p* *=* *0.50, 0.98*) were then considered for TDR analysis. For simplicity the authors refer herein to tumor-dose-rate always as TDR_s,p_ in combination with time span *s* and PTV percentage *p* of specific brain metastases. For all TDR_s,p_ with time spans *s* > *T* they specified:

4 $$\mathrm{TDR}_{\mathrm{s}>\mathrm{T},\mathrm{p}}=\mathrm{TDR}_{\mathrm{T},\mathrm{p}}$$

### Technical and clinical treatment parameters

The authors analyzed TDR_s,p_ dependence on: (a) treatment time and sub-parameter (treated lesions, beam directions, beams/metastasis and monitor units [MU]), (b) prescription dose and sub-parameter (absolute dose, prescription isodose and maximum dose) and (c) metastasis properties and sub-parameter (total or single metastasis volume and average radiologic depth/beam to metastasis). Furthermore, two parameters were introduced to describe the relation of the selected collimators to each treated metastasis. A volume parameter was specified according to the effective dose sphere of each collimator based on its diameter in the center of the metastasis treated in relation to the PTV and defined *w*_*Volume*_ as:

5 $$w_{\text{Volume}}=\frac{PTV}{\sum _{c}\left(\frac{\pi {d_{c}}^{3}}{6}\right)}$$

where *c* is the collimator targeting the PTV with diameter d_c_ (in millimeters). Furthermore, a treatment planning parameter was specified according to the mean number of lesions targeted per collimator for collimators *c* targeting the specific PTV and defined *w*_*Collimator*_ as:

6 $$w_{\text{Collimator}}=\frac{\sum _{c}\left(|\mathrm{PTV}_{c}|\right)}{|c|}$$

where *|PTV*_*c*_*|* is the number of PTVs targeted by collimator *c* and *|c|* is the number of collimators used for the PTV. In order to correlate TDR_s,p_ to clinical outcome, TDR_s,p_ combinations were analyzed as described above in relation to the RECIST (Response Evaluation Criteria in Solid Tumors) classifications during first (3 months) and overall follow-up under consideration of clinical parameters potentially influencing local control such as simultaneous TT/IT or chemotherapy, prior WBRT and dose [8, 27, 28].

### Follow-up and statistical analysis

All patients received magnetic resonance imaging (MRI) identical to treatment planning according to best practice at 6–8 weeks and 3/6/9/12 months after treatment and every 6 months thereafter [1]. For this work, the final follow-up was performed 04/2019 to capture long-term TDR effects. Local response assessment was performed using RECIST, classified into complete response (CR), partial response (PR), local stable disease (SD) and local progressive disease (PD). Local response assessment included the differentiation of PD and radiation necrosis (RN) according to standard practice [27].

Local control (LC) and overall survival (OS) were estimated using the Kaplan-Meier method with SPSS (v20.0, IBM, Armonk, USA). For modeling dependencies between TDR and plan parameters, Spearman’s rank correlation coefficients (ρ) using SPSS and coefficients of determination (R^2^) from linear and power regression using Excel (v2007, Microsoft, Seattle, USA) were calculated. Univariate analyses using Cox proportional hazard regression models were performed to investigate the patient disease characteristics and dosimetric parameters as predictors of OS, LC (CR or PR or SD), local tumor response (CR or PR) and RN. For Cox regression censoring was done at last follow-up and for LC, local tumor response and RN also at time of death. Stepwise forward conditional methods were further performed in multivariate analysis incorporating variables that were found to be significant (*p* ≤ 0.05) in univariate analyses.

## Results

### Tumor-dose-rate results

**Fig. 1:**
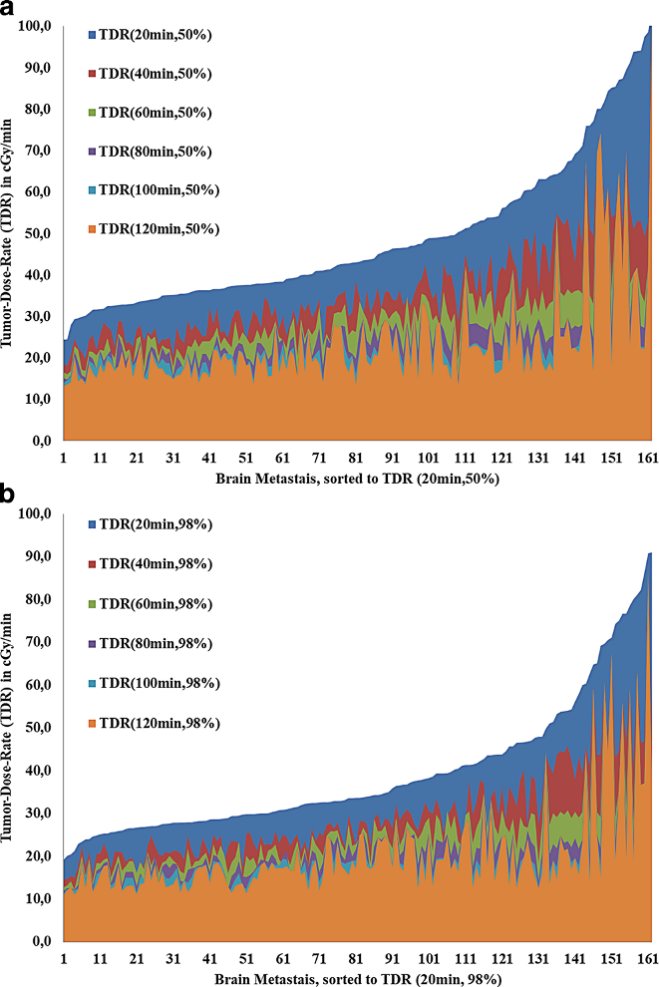
Overlay plot of tumor-dose-rates (*TDR*) with variable time span *s* for planning target volume percentages 50% (**a**) and 98% (**b**). Large differences can be seen for TDR_20min,X%_, with highly variable and unpredictable TDR reductions for longer time spans

The TDR_s,p_ was mainly dependent on time span *s* rather than on PTV percentage *p* (Fig. 1) and varied strongly between patients and metastases within the same patient. The median TDR_s,p_ decreased from 0.34 Gy/min (0.19–0.91 Gy/min) for TDR_20min,98%_ to 0.17 Gy/min (0.11–0.86 Gy/min) for TDR_120min,98%_, while the difference between median TDR_s,50%_ and TDR_s,98%_ was reduced from 0.09 Gy/min for TDR_20min,p_ to 0.03 Gy/min for TDR_120min,p_. The overall median TDR_60min,98%_ was 0.23 Gy/min (0.12–0.87 Gy/min) while the median TDR_60min,98%_ was 0.22 Gy/min (0.12–0.63 Gy/min) for MBM and 0.30 Gy/min (0.23–0.87 Gy/min) for OBM, respectively.

### Treatment parameter correlation

**Fig. 2:**
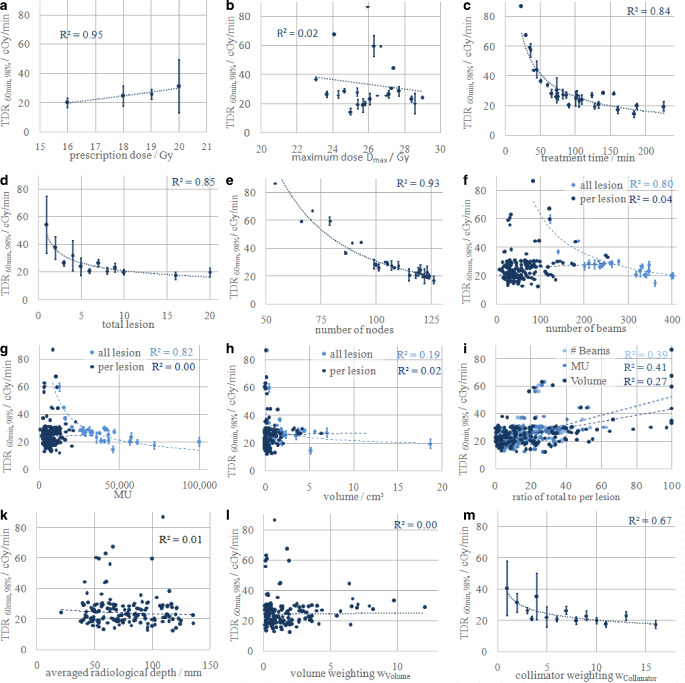
Relationship of median tumor-dose-rate (*TDR*_*60min,98%*_) and **a** prescription dose, **b** maximum dose, **c** treatment time, **d** lesions per session, **e** number of beam directions (nodes), **f** number of beams, **g** monitor units (*MU*), **h** treated volume, **i** ratio of the number of beams, MU and volume to the plan total value, **k** averaged radiological depth of the beams to metastasis, **l** volume factor *w*_*volume*_ and **m** collimator factor *w*_*collimator*_. Also shown are linear and power regressions and their resulting coefficients of determination R^2^

TDR_s,50%_ correlated slightly better with all parameters than TDR_s,98%_ (mean ρ = 0.422 vs. ρ = 0.380) and the mean correlation increased between *s* *=* *20–80* *min*. For data compression the authors present TDR_60min,98%_ as an example, which showed the highest correlation over all parameters. They noticed slight linear mean increases of 0.03 Gy/min per prescribed Gray (Fig. 2a, R^2^ = 0.95), although TDR_60min,98%_ was not affected by maximum dose (Fig. 2b, R^2^ = 0.02 and ρ = −0.037 with *p* = 0.636). Furthermore, they found strong correlations between TDR_60min,98%_ and treatment time (Fig. 2c, R^2^ = 0.84 and ρ = −0.717 with *p* < 0.001) with a plateau of 0.22 Gy/min with *s* *≥* *80* *min*. The sub-parameter also correlated well with MU (Fig. 2g, R^2^ = 0.82 and ρ = −0.767 with *p* < 0.001), number of beams (Fig. 2f, R^2^ = 0.80 and ρ = −0.755, *p* < 0.001) and beam directions (Fig. 2e, R^2^ = 0.93 and ρ = −0.685, *p* < 0.001). For the number of beam directions the authors saw strong differences for the TDR between <95 and 95–110 and >110 beam directions. Furthermore, they noticed strong correlations between TDR_60min,98%_ and the number of treated lesions with notable mean TDR_60min,98%_ decrease for OBM and for MBM between ≤4 and >4 metastases (Fig. 2d, R^2^ = 0.85).

Otherwise, the cumulative and single PTV, number of beams and MU per lesion did not correlate with TDR_60min,98%_ (Fig. 2f–h, R^2^ ≤ 0.05 and ρ = 0.022–0.263, *p* = 0.001–0.782), while the ratio of the values per lesion to the cumulative values correlated weakly (Fig. 2i and ρ = 0.358/0.546/0.492, *p* < 0.001). Furthermore, neither the averaged radiological beam depth nor *w*_*Volume*_ correlated with TDR_60min,98%_ (Fig. 2k/l and ρ = −0.067/0.139, *p* = 0.078/0.398). Lastly, *w*_*Collimator*_ correlated well with TDR_60min,98%_ (Fig. 2m and ρ = −0.708, *P* < 0.001) and notable mean TDR decrease between ≤4 and >4 lesions per collimator was observed.

### Clinical outcome correlation

**Fig. 3:**
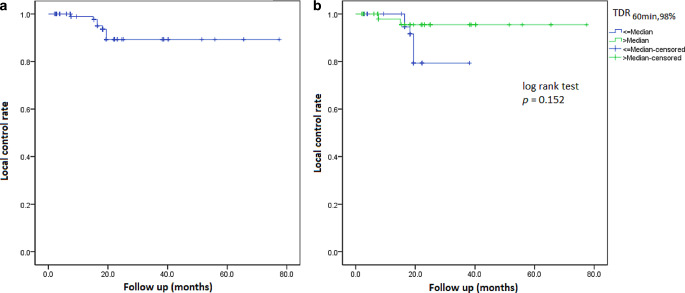
Kaplan-Meier curves censored for overall local control (**a**) and local control split based on median tumor-dose-rate (TDR_60min,98%_) (**b**)

Median OS was 20 months and OS at 1 and 2 years was 60.6% and 38.7%, respectively. NSCLC patients had the worst OS (HR = 3.152, CI = 1.937–5.129, *p* < 0.001). Five MBM patients with 25 metastases could not be radiologically evaluated due to early demise after SRS. Otherwise, median follow-up was 17.3 months (2.3–40.2 months) for MBM and 38.2 months (4.3–77.5 months) for OBM, respectively. Overall crude and actuarial 1‑year and 2‑year LC was 94.9%, 98.8% and 90.3%, respectively (Fig. 3), with one field-border recurrence for OBM (5.3% of 19 lesions) and one field-border and five in-field recurrences for MBM (5.1% of 118 lesions). No correlation was found between any parameter, including TDR, and LC (Table 2), although the events are limited.

**Table 2:** Univariate and multivariate analysis

*All parameters* *≥* *vs. < median if not indicated otherwise*	Local control Univariate analysis Cox regression		Local response Univariate analysis Cox regression		Local response Multivariate analysis Cox regression		Local tissue reaction Univariate analysis Cox regression	
	HR (CI)	*p*-Value	HR (CI)	*p*-Value	HR (CI)	*p*-Value	HR (CI)	*p*-Value
TDR_(20_ _min,98%)_	0.976 (0.917–1.039)	0.440	0.982 (0.967–0.997)	0.019*	0.981 (0.864–1.113)	0.762	1.011 (0.981–1.042)	0.468
TDR_(20_ _min,50%)_	0.983 (0.933–1.035)	0.510	0.982 (0.968–0.995)	0.009*	0.975 (0.881–1.080)	0.633	1.014 (0.987–1.041)	0.327
TDR_(40_ _min,98%)_	0.938 (0.837–1.050)	0.265	0.974 (0.954–0.995)	0.014*	1.097 (0.878–1.371)	0.414	1.009 (0.970–1.050)	0.663
TDR_(40_ _min.50%)_	0.949 (0.864–1.041)	0.267	0.972 (0.953–0.992)	0.006*	0.959 (0.804–1.144)	0.642	1.011 (0.976–1.048)	0.532
TDR_(60_ _min.98%)_	0.925 (0.809–1.059)	0.261	0.974 (0.952–0.996)	0.019*	1.041 (0.764–1.418)	0.800	1.004 (0.964–1.046)	0.835
TDR_(60_ _min.50%)_	0.940 (0.841–1.051)	0.275	0.973 (0.953–0.994)	0.011*	0.939 (0.726–1.213)	0.628	1.008 (0.973–1.044)	0.672
TDR_(80_ _min.98%)_	0.924 (0.803–1.065)	0.275	0.976 (0.956–0.997)	0.023*	0.960 (0.639–1.441)	0.844	1.007 (0.970–1.045)	0.726
TDR_(80_ _min.50%)_	0.946 (0.851–1.052)	0.305	0.978 (0.960–0.996)	0.019*	0.990 (0.833–1.176)	0.910	1.010 (0.979–1.042)	0.539
TDR_(100_ _min.98%)_	0.949 (0.852–1.058)	0.344	0.978 (0.958–0.997)	0.025*	0.680 (0.169–2.735)	0.587	1.005 (0.969–1.043)	0.772
TDR_(100_ _min.50%)_	0.956 (0.871–1.050)	0.350	0.979 (0.961–0.997)	0.020*	1.127 (0.366–3.467)	0.835	1.008 (0.977–1.039)	0.619
TDR_(120_ _min.98%)_	0.957 (0.871–1.051)	0.362	0.979 (0.961–0.998)	0.029*	1.440 (0.415–4.991)	0.566	1.004 (0.968–1.041)	0.839
TDR_(120_ _min.50%)_	0.964 (0.889–1.045)	0.368	0.980 (0.964–0.997)	0.023*	0.955 (0.316–2.881)	0.934	1.006 (0.976–1.038)	0.696
PTV	1.000 (0.999–1.001)	0.775	1.000 (0.999–1.000)	0.069	–	–	1.001 (1.0005–1.0012)	<0.001*
PTV D_98%_	1.007 (0.563–1.799)	0.982	0.796 (0.682–0.930)	0.004*	0.873 (0.445–1.710)	0.692	0.830 (0.579–1.190)	0.311
PTV D_Max_	0.928 (0.535–1.607)	0.788	0.837 (0.712–0.984)	0.032*	0.669 (0.423–1.057)	0.85	0.696 (0.469–1.035)	0.073
WBRT (prior vs. none)	2.452 (0.269–22.370)	0.427	1.262 (0.798–1.997)	0.319	–	–	1.555 (0.410–5.901)	0.516
Histology (NSCLC vs. MLA)	5.903 (0.672–51.848)	0.109	3.655 (1.507–8.864)	0.004*	4.010 (0.903–17.814)	0.068	0.260 (0.055–1.233)	0.090
Histology (BC vs. MLA)	2.602 (0.141–48.150)	0.521	3.375 (1.358–8.389)	0.009*	4.847 (1.131–20.779)	0.034*	0.403 (0.088–1.838)	0.240
Conservative therapy (chemo vs. none)	11.630 (1.347–100.39)	0.026	0.970 (0.533–1.763)	0.919	–	–	2.930 (0.856–10.026)	0.087
Conservative therapy (TT/IT vs. none)	<0.001 (Infinity)	0.968	0.323 (0.152–0.687)	0.003*	0.175 (0.039–0.800)	0.025*	0.590 (0.068–5.126)	0.633

*TDR* Tumor-dose-rate, *PTV* planning target volume, *WBRT* whole brain radiotherapy, *NSCLC* non-small-cell lung cancer, *MLA* melanoma, *BC* breast cancer, Chemo chemotherapy, *TT/IT* targeted therapy/immunotherapy, *HR* hazard ratio, *CI* confidence Interval

*Statistical significance

Local response as defined by CR/PR at first (6–8 weeks after SRS) and any follow-up were 47.5% and 61.0% for MBM and notably higher with 68.4% and 73.7% for OBM, respectively. Local responses at any follow-up for SRS with concomitant TT/IT, chemotherapy or no therapy was 81.7%, 54.2% and 35.7%, respectively. For overall follow-up, mean TDR_60min,98%_ was higher with 0.28 ± 0.17 Gy/min for responding (CR/PR) vs. 0.23 ± 0.12 Gy/min for non-responding (SD/P) metastases, although mean TDR_60min,98%_ was not notably different in the sub-groups (CR = 0.27, PR = 0.31, SD = 0.22, *P* = 0.24 Gy/min). Univariate analysis showed significant correlations for local response with almost any TDR, but also with dose (D_max_ and D_98%_), concomitant therapy and primary tumor histology (Table 2). In multivariate analysis concomitant therapy with TT/IT and breast cancer as primary histology remained significant.

RN (grade ≥2) occurred for six metastases (5.2% of 118 lesions) for MBM and for five metastases (26.3% of 19 lesions) for OBM, although there was no relevant difference for TDR_60min,98%_ between the RN (0.27 ± 0.07 Gy/min) and non-RN (0.26 ± 0.12 Gy/min) group. The statistical analysis showed only target volume as a significant factor for RN (Table 2), albeit with small relative effects (HR = 1.001, CI = 1.0005–1.0012, *p* < 0.001). Three of the local reactions (two in one patient simultaneously) needed to be operated, but otherwise other grade ≥3 side effects were not noticed.

## Discussion

For the first time, the authors demonstrated high local TDR variations for CyberKnife stereotactic radiosurgery. They found that maximum dose delivered over certain time frames during treatment is notably lower for multiple (>3) brain metastases as compared to oligo (1–3) brain metastases. This may translate into the possibility for some tumor cells to repair DNA damage during prolonged treatment and to re-populate to form local tumor recurrences. While DNA repair in prolonged dose delivery is well described for normal cell types [15, 16], *in-vitro* studies for tumor cell lines showed none or even opposite effects [17, 18]. On the other hand, BED simulations which included treatment time components have shown negative impacts of more complex treatment plans and hence prolonged treatment on tumor control [19, 20].

However, all studies lacked clinical outcome data correlation and the authors found that the *in-vivo* response of brain metastases after SRS is notably different if the TDR during treatment is lower. Explicitly for locally responding metastases (complete/partial remission) they found higher mean TDR_60min,98%_ of 0.28 ± 0.17 Gy/min vs. 0.23 ± 0.12 Gy/min for non-responding metastases (local stable/progressive disease). This difference was similar in short- and long-term response. However, while their analysis may point to clinical influences of TDRs during treatment, they were not able to directly correlate low TDR with sole local progression due to low recurrence rates in their cohort. To some extent, this might be explained by shorter follow-up in the MBM group. The results may also be influenced by concurrent medication where the combination with TT/IT showed higher response rates as compared with concurrent chemotherapy or no concurrent medication. The synergistic effects of combined SRS and TT/IT are well described [8–11, 28, 29], although local response is often not presented in greater detail. Additionally, local control is often correlated to dose and lesion volume, but the authors did not see any dose or lesion and/or volume based response differences in their analysis, which might be explained by GTV mean dose optimization [24, 30, 31].

Technically, the TDR variations were also not correlated with lesion volume(s), depth of the lesion or any volumetric ratio or parameter. This may point to the possibility that the TDR can be kept constant with various collimators regardless of lesion size and location, limiting this statement to the lesion volumes presented in this work (0.01–4.64 cc). On the other hand, TDR variations were mainly driven by treatment time and each sub-factor like number of treated lesions, beams and beam directions as well as MU. This comes as no surprise as the CyberKnife treatment time can be split into equal thirds between imaging, robot motion and beam delivery time [21], which directly correlates to number of beams, directions and MU, respectively. Especially the number of beam directions (robot motion time), even with optimized robot path traversal, is highly correlated to TDR with strong differences between <95 and 95–110 and >110 directions. However, a large number of directions and beams are needed in order to avoid hotspots between lesions and to ensure low exposure of healthy brain tissue [23, 32–34], and a further increase in the CyberKnife working space [35] will only worsen this specific problem. One possible solution may be reduced imaging frequency, although the authors already adapted the frequency to the patient position stability during treatment and a further decrease may significantly affect treatment accuracy [36]. Another solution may come from increased linear accelerator dose-rates, but a change from our 800 MU/min linear accelerator to 1000 MU/min would have only resulted in a 4-min averaged treatment time reduction and a further increase in MU/min is currently not feasible for the small accelerator head.

Surely, one may switch altogether to different platforms with much higher dose-rates, but the in-treatment accuracy and possible dose gradients for intracranial SRS appear to still be inferior with c‑arm based linear accelerators as compared to dedicated SRS systems [12, 23, 33, 34]. On the other hand, c‑arm based systems are capable of delivering dose during gantry rotation, which decreases treatment time tremendously. The newer CyberKnife version may also enable this option [37], although it seems questionable whether this technique is feasible for multiple lesions treated simultaneously and further developments are surely necessary. Additionally, the use of multi-leaf-collimators for the CyberKnife (InCise, Accuray) [38] may not result in adequate dose distributions for multiple small metastases as compared to cylindrical cones despite significantly reduced treatment times [39]. Furthermore, not even the use of the dynamic Iris collimator (Accuray) [40] may be able to resolve TDR variations as the necessary MU will be higher due lower output factors for the smallest field sizes [41], and the beam repositioning towards multiple lesions in every direction may obliterate the benefits of reduced robot motion time with a single collimator, pending further investigation. Additionally, most metastases in this evaluation were targeted by the 5‑mm fixed collimator and for this field size the IRIS collimator is not advisable due to field size reproducibility concerns.

A real reduction of TDR variations may be achieved by smart collimator selection and plan splitting based on geometric regions of the metastases. The present results indicate that the use of single collimators for >4 lesions will notably decrease TDR. Since multiple collimators are beneficial for plan quality [25], the collimator selection and delivery sequence could be optimized. However, this has already been performed in the present cohort to the extent possible and since the smallest field size (5 mm) is used for most lesions this option may be limited. On the other hand, plan splitting with only few brain metastases per plan or even per day may be feasible [42], and the authors now use this possibility whenever the integral brain dose does not unreasonably increase. Other methods for increasing TDR may come from increased prescription dose, which is of course bound to healthy brain dose limits, and from fractionation based on isotoxic planning [43], although this would only reduce the actual MU per session and hence affect only one third of the treatment time. A truly viable solution to the problem of low TDR during treatment may therefore only come from sequential beam delivery per actual treated target [21]. However, this will of course increase the overall treatment time due to the higher robot travel required, which could then be countered by dose delivery during robot motion as already discussed [37].

Limitations to the present analysis include the sample size (23 patients with 162 brain metastases), especially in the sub-groups, the mono-centric and mono-planner perspective, even though planning was based on international best practice guidelines, and the retrospective nature of the clinical response evaluation, even though the authorsʼ database was designed as a prospective register. Further limitations are bound to the nature of the patients’ diseases and the resulting limited life expectancy after developing multiple brain metastases. Hence, the statistical analysis for local control and response may be influenced by high censoring due to patientsʼ early demise. In the future and with higher sample sizes, the use of cumulative incidence functions under considerations of competing risks may be considered for analysis instead of the generally widely used Cox regression. Finally, further prospective analysis within multi-center cohorts which are based on the suggested optimization strategies for low TDR are necessary.

## Conclusion

Large TDR variations are noted during robotic SRS of multiple brain metastases which mainly arise from prolonged treatment times due to treatment complexity. Clinically, low TDRs corresponded with decreased local tumor responses, although the main influencing factor was concomitant medication. Optimization of the TDR may solely come from sequential beams per lesion dose application, pending further investigation in multi-center cohorts.
